# A Comparative Analysis on the Innate Immune Responses of *Cirrhinus mrigala* Challenged with *Pseudomonas aeruginosa* and *Fusarium oxysporum*

**DOI:** 10.3390/ijms241512392

**Published:** 2023-08-03

**Authors:** Zaeema Usman, Zakia Kanwal, Asima Tayyeb, Iqra Noshair, Imran Haider, Naushad Ahmad, Suliman Yousef Alomar

**Affiliations:** 1 Department of Zoology, Faculty of Natural Sciences, Lahore College for Women University, Lahore 54000, Pakistan; zaeemausman@gmail.com (Z.U.); iqra.noshair@lcwu.edu.pk (I.N.); 2 School of Biological Sciences, University of the Punjab, Quaid-e-Azam Campus, Lahore 54590, Pakistan; asima.sbs@pu.edu.pk; 3 Swammerdam Institute for Life Sciences, University of Amsterdam, 1012 Amsterdam, The Netherlands; i.haider@uva.nl; 4 Department of Chemistry, College of Science, King Saud University, Riyadh 11451, Saudi Arabia; anaushad@ksu.edu.sa; 5 Zoology Department, College of Science, King Saud University, Riyadh 11451, Saudi Arabia; syalomar@ksu.edu.sa

**Keywords:** bacterial infection, *Pseudomonas aeruginosa*, *Fusarium oxysporum*, *Cirrhinus mrigala*, gene expression, immunology

## Abstract

Microbes are the most significant ubiquitous pathogens that cause serious infections in freshwater fish, leading to tremendous economic losses. The present study was designed to investigate the extent of changes in cytokine expression, hemato-biochemical parameters, and tissue histology of *Cirrhinus mrigala* (*C. mrigala*) challenged with *Pseudomonas aeruginosa* (*P. aeruginosa*) and *Fusarium oxysporum* (*F. oxysporum*). Fish were divided into three major groups: control, *P. aeruginosa*-challenged, and *F. oxysporum*-challenged. The infection in both challenge assays was allowed to progress until 7 days post infection. Upregulated expression of *TNF-α* and *IL-1β* was found in blood, gills, livers, and kidneys of the challenged fish. Significant differences were noted in hematological parameters of challenged fish. Alanine aminotransferase, aspartate aminotransferase, and alkaline aminotransferase levels also showed significant differences in infected and control groups. An increase in serum albumin and globulin and a decrease in total protein were noted in infected groups as compared to the control group. Severe histological alterations were noted in gill, liver, and kidney tissues of the infected groups as compared to control. The order of histological alteration index for *P. aeruginosa* challenge was liver > kidney > gills, and for *F. oxysporum* challenge it was kidney > liver > gills. These changes in fish infected by *P. aeruginosa* and *F. oxysporum* can be used as an effective and subtle index to monitor the physiological and pathological conditions of fish.

## 1. Introduction

Carp culture faces an alarming condition as a consequence of pathogenic challenges, resulting in an increased infection rate and mortalities due to loss of immune function [1]. Some of the key factors such as stress, contaminated diet, and unsanitary conditions contribute to an epidemic of bacterial infections in aquatic animals [2]. Disease outbreaks resulting from infections in fish pose a significant challenge to the aquaculture industry as they can lead to substantial economic losses due to illness. According to estimates, up to 50% of farmed fish die due to infections before being marketed. The major attributes of the economic losses are considered to be poor growth, excessive mortalities, and poor fish quality. High-density fish rearing exacerbates the transmission and proliferation of microorganisms [3,4]. Bacteria, viruses, parasites, and biological toxins are the most common hazards in aquaculture systems [5,6]. From the epizootiological point of view, bacterial diseases are more common than parasitic and viral diseases [7,8]. Furthermore, most outbreaks are attributed to bacterial causes that are devastating to the aquaculture system such as *Aeromonas hydrophila, Flavobacterium columnare, Pseudomonas* spp., *Streptococcus* spp., *Proteus* spp, *Enterococcus* spp., *Citrobacter freundii*, and *Yersinia ruckeri* [9].

Pathogens directly or indirectly affect fish health and limit physiological responses. Infection caused by *Pseudomonas aeruginosa* (*P. aeruginosa*) in fish leads to the development of a condition known as red skin illness, which occurs throughout the year, particularly when fish are injured due to improper handling or during transportation [10]. Fungal infections pose significant threats to aquaculture, inflicting long-lasting damages. *Fussarium oxysporum (F. oxysporum*) has emerged as a prominent fish pathogen, causing acute infections in fish, and lack of effective disease control methods contributes to elevated mortality rates [10]. Microbial infections not only cause external phenotypic changes in infected fish but also lead to significant alterations in liver enzyme activities and hematological indices [11,12,13].

Evaluating cytokine responses, which develop in the early stages of infections, is essential in determining the behavior of both host and pathogen during and after the infection. During infections, the secretion of cytokines such as interleukins, interferons, and tumor necrosis factors hold great importance [14]. The mRNA levels of interleukin-1β (*IL-1β*) and tumor necrosis factor alpha (*TNF-α*) significantly change in response to bacterial infections, indicating their association with other immune-related molecules in fish during infections [15]. 

Blood parameters have been frequently used as pathophysiological indicators to assess the structural and functional condition of fish exposed to a wide range of toxins [15,16]. Analysis of serum biochemical parameters is particularly beneficial for identifying target organs and the general health of animals, and it is encouraged for providing early indicators of different changes in stress conditions [17]. Histological techniques are useful in assessing the sublethal effects of pollutants or pathogens, and pathological alterations can function as biomarkers. Furthermore, histological changes offer a quick, intermediate way to identify stressors and detect their effects on tissues [18,19].

In the present study, we challenged *Cirrhinus mrigala* (*C. mrigala*) with *P. aeruginosa* and *F. oxysporum* to investigate the changes in fish blood physiology, biochemical response, histopathology, and inflammatory gene expression. Therefore, this study provides novel insights into innovative prophylactic measures and effective strategies to combat microbial challenges in aquaculture.

## 2. Results

### 2.1. Behavior and Phenotypic Alterations 

The behavior of fish in the control and challenged groups was observed twice daily (Table 1). Control fish moved actively in the aquarium throughout the observing period. In control, fin movements and response of the fish to feed was normal. No specific aggressive interaction was noted in control fish. However, the activity of *P. aeruginosa*-challenged fish was substantially decreased. It is noteworthy that *P. aeruginosa*-challenged fish showed slower swimming movements. Fish were seen to gather in corners of the aquarium and near the oxygen-inlet points. Fish challenged with *F. oxysporum* showed impaired competitive foraging ability, lethargy, restlessness, and slow fin movement and attempted to jump out of the aquaria and were gasping at the surface of the water. By 7 dpi, complete rejection of the feed and unbalanced swimming was observed in the *F. oxysporum*-challenged group.

**Figure 1:**
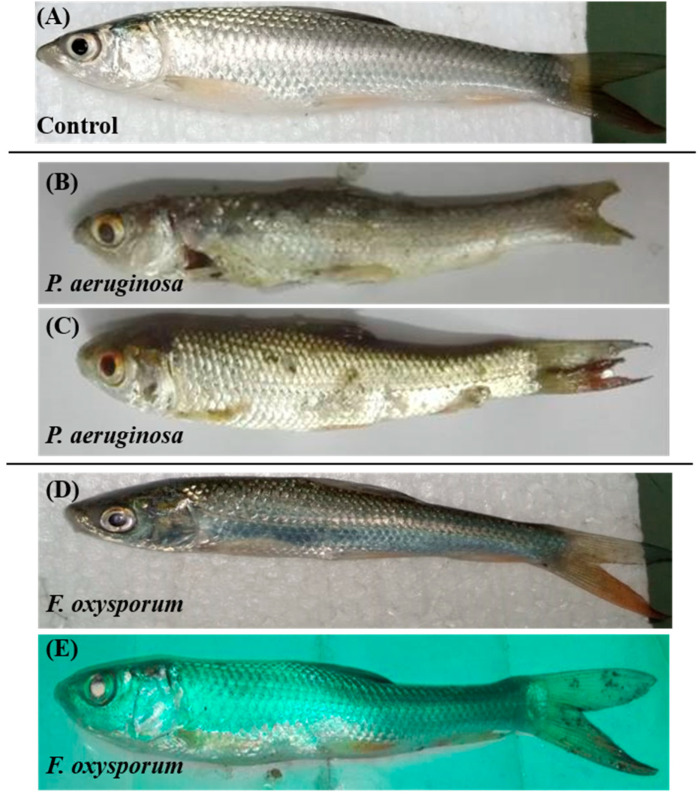
*Pseudomonas aeruginosa* (*P. aeruginosa)* and *Fussarium oxysporum* (*F. oxysporum*) infection progression in *C. mrigala* (n = 15). (**A**) Control fish head, trunk region, and tail region. (**B**,**C**) *P. aeruginosa-*infected fish showed eye redness and gill damage, hemorrhage, ulcerative lesions, swelling, scale loss, and tail rupturing. (**D**,**E**) *F. oxysporum*-infected fish showed redness of eye, skin ulcerations at tail, inflammation, exophthalmia, and pale color of gills.

Fish were also examined for gross pathological changes. In control fish, no pathological changes appeared (Figure 1A). In the *P. aeruginosa-*challenged group, fish showed pathological symptoms starting from 5 dpi, characterized by eye redness and gill damage, hemorrhage, ulcerative lesions, swelling, scale loss, and tail rupturing (Figure 1B,C). Fish challenged with *F. oxysporum* showed discoloration of body, skin ulcerations at the tail, inflammation, exophthalmia, and pale color of gills starting from 3 dpi (Figure 1D,E).

### 2.2. Cytokine Expression Analysis

**Figure 2:**
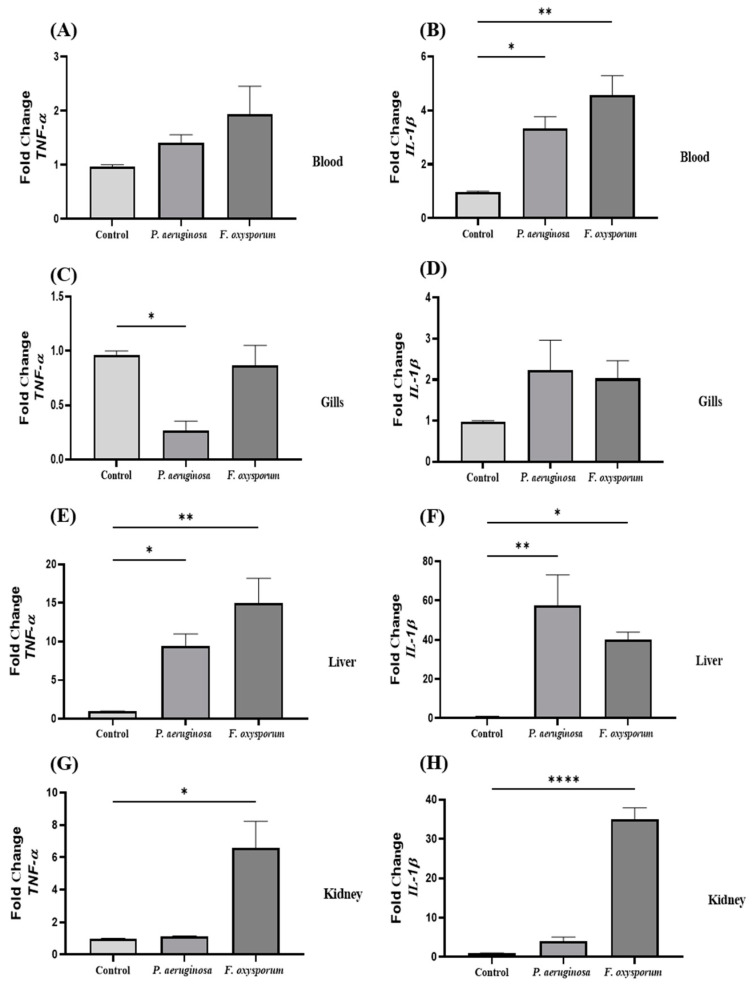
Gene expression analysis of *TNF-α* and *IL-1β* in control, *P. aeruginosa-,* and *F. oxysporum*-challenged groups at 7 dpi in blood, gills, liver, and kidney tissues (**A**–**H**, respectively) of *C. mrigala* (n = 15). Data are presented as mean ± SEM (* *p* < 0.05; ** *p* < 0.01; **** *p* < 0.0001).

Modulation in differential expression of *TNF-α* and *IL-1β* in blood, gills, liver, and kidney were analysed using qRT-PCR. *TNF-α* expression was increased in blood of *P. aeruginosa-* (≈1.4-fold) and *F. oxysporum*-challenged fish *(*≈1.9-fold) as compared to control (Figure 2A). The *IL-1β* gene transcript level in blood was significantly upregulated in the *F. oxysporum* group (≈4.56-fold) and *P. aeruginosa* (≈3.33-fold) as compared to the control group (Figure 2B). In gills, *TNF-α* was significantly downregulated in *P. aeruginosa* (≈0.26-fold) and upregulated in the *F. oxysporum* (≈0.87-fold) group as compared to the control group (Figure 2C). Likewise, expression of *IL-1β* in gills was increased in the *P. aeruginosa* (≈2.23-fold) and *F. oxysporum* (≈2.03-fold) groups in contrast to the control group (Figure 2D). Similarly, in livers, *TNF-α* and *IL-1β* expression was significantly elevated in the *P. aeruginosa* (≈9.46-fold, ≈57.6-fold) and *F. oxysporum* (≈14.96-fold, ≈39.9-fold) groups relative to the control group (Figure 2E,F). In kidneys, increased expression of *TNF-α* was recorded in the *F. oxysporum* (≈6.56-fold) group and *P. aeruginosa (*≈1.1-fold) as compared to the control group (Figure 2G). Upregulated expression of *IL-1β* was noted in *F. oxysporum* (≈35.1-fold) and *P. aeruginosa* (≈3.96-fold) groups as compared to the control group (Figure 2H).

### 2.3. Hematological Analysis

**Figure 3:**
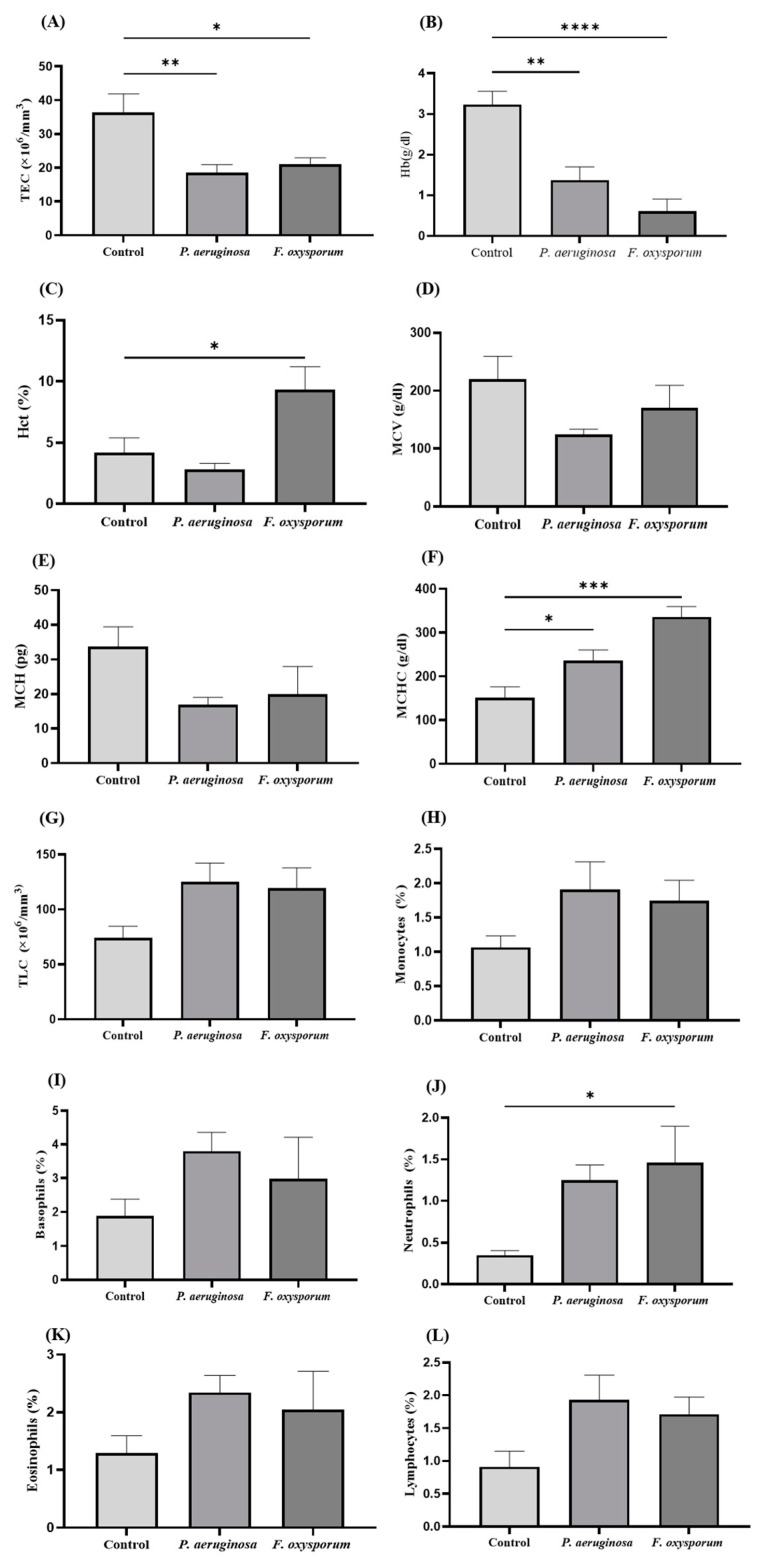
Evaluation of hematological parameters in control, *P. aeruginosa-,* and *F. oxysporum*-challenged groups (n = 15) at 7 dpi. (**A**) Total erythrocyte count (TEC), (**B**) Hemoglobin (Hb), (**C**) Hematocrit (Hct%), (**D**) Mean corpuscular volume (MCV), (**E**) Mean corpuscular hemoglobin (MCH), (**F**) Mean corpuscular hemoglobin concentration (MCHC), (**G**) Total leukocyte count (TLC), (**H**) Monocytes (%), (**I**) Basophils (%), (**J**) Neutrophils (%), (**K**) Eosnophils (%), and (**L**) Lymphocytes (%). Data are presented as mean ± SEM (* *p* < 0.05; ** *p* < 0.01; *** *p* < 0.001; **** *p* < 0.0001).

Results of the present study showed a marked decrease in TEC, Hb, Hct, MCV, MCH, and MCHC concentration levels in *P. aeruginosa-* and *F. oxysporum-*challenged groups compared to the control (Figure 3A–F, respectively). Likewise, TLC level was noted as being higher in *P. aeruginosa-* and *F. oxysporum*-infected fish in relation to control (Figure 3G). Differential Leukocyte Count analysis showed an increase in the number of monocytes, basophils, neutrophils, eosinophils, and lymphocytes in the *P. aeruginosa* and *F. oxysporum* groups than the control group (Figure 3H–L).

### 2.4. Biochemical Analysis 

**Figure 4:**
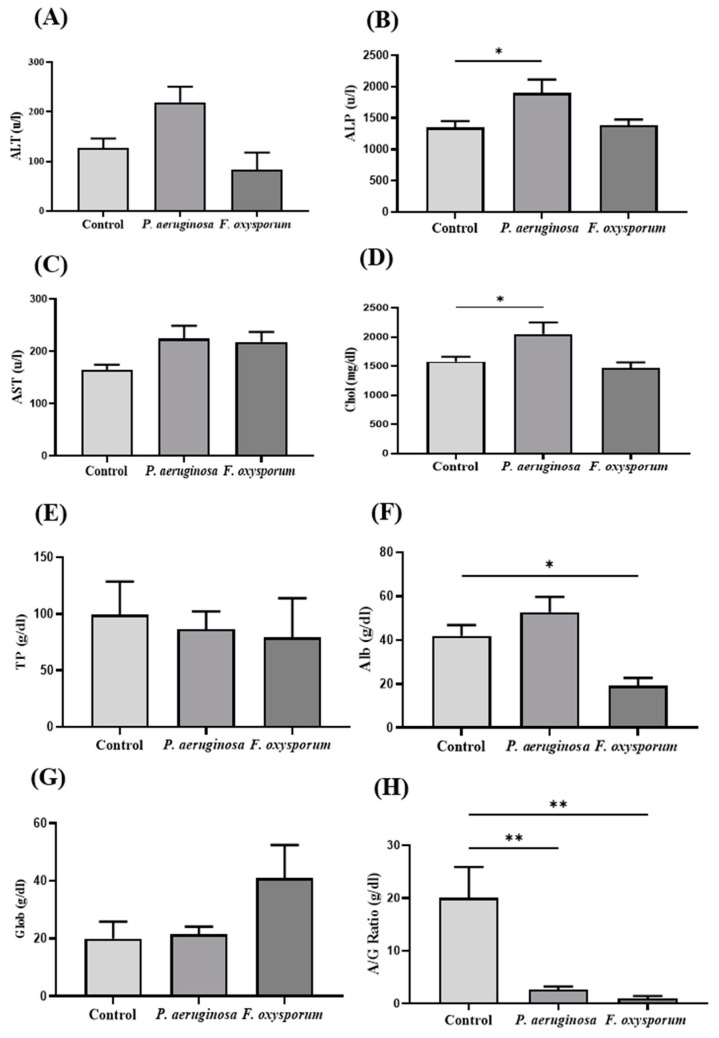
Biochemical parameters in control, *P. aeruginosa-*, and *F. oxysporum*-challenged groups *(*n = 15) at 7 dpi. (**A**) Alanine aminotransferase (ALT), (**B**) Alkaline phosphatase (ALP), (**C**) Aspartate aminotransferase (AST), (**D**) Cholesterol (Cho), (**E**) Total Protein (TP), (**F**) Albumin (Alb), (**G**) Globulin (Glob), and (**H**) A/G ratio. Data are presented as mean ± SEM (* *p* < 0.05; ** *p* < 0.01).

Enzymes ALT and ALP showed a significant increase in their induction after 7 dpi in the *P. aeruginosa* group, and they decreased in *F. oxysporum-*challenged groups as compared to the control group (Figure 4A,B). The AST level was increased in *P. aeruginosa-* and *F. oxysporum-*challenged groups as compared to the control group (Figure 4C). Cho level was significantly increased (*p < 0.05*) in the *P. aeruginosa-*challenged group as compared to the *F. oxysporum-*challenged and control groups (Figure 4D). The level of TP decreased in *P. aeruginosa-* and *F. oxysporum-*challenged groups compared to the control group (Figure 4E). Alb was decreased significantly (*p < 0.05*) in the *F. oxysporum-*challenged group, and it was increased in the *P. aeruginosa* group as compared to the control (Figure 4F). Glob level was observed to have increased in *F. oxysporum-* as compared to *P. aeruginosa-*challenged and control groups (Figure 4G). A/G ratio found to be significantly decreased in *P. aeruginosa-* and *F. oxysporum-*challenged groups relative to the control group (Figure 4H). 

### 2.5. Histological Examination 

**Figure 5:**
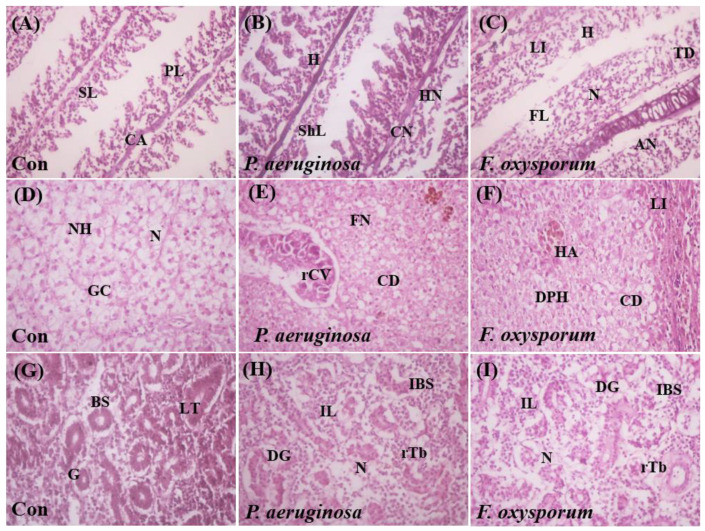
Histological evaluation of target tissues of *C. mrigala* (n = 15). Gill tissues of control, *P. aeruginosa-* and *F. oxysporum*-challenged groups (**A**–**C**), respectively. Primary lamellae (PL), Secondary lamellae (SL), Central axis (CA), Tissue debris (TD), Hemorrhage (H), Cellular necrosis (CN), Hypertrophy of nuclei (HN), Fusion of lamellae (FL), Shortening of lamellae (ShL), Leukocyte infiltration (LI), Aneurism (AN). Liver tissues of control, *P. aeruginosa-,* and *F. oxysporum*-infected fish (**D**–**F**), respectively. Hepatocytes (H), Cytoplasmic degeneration (CD), Rupturing of the central vein (rCV), Fibronectin (FN), Granulated cytoplasm (GC), Nuclear hypertrophy (NH), Hepatic abscess (HA), Degeneration of pancreatic hepatocytes (DPH). Kidney tissues of control, *P. aeruginosa-,* and *F. oxysporum*-infected fish (**G**–**I**), respectively. Glomerulus (G), Bowman’s space (BS), Fine renal tubules (rTb), Inflammation (I), Necrosis (N), Longitudinal section of tubule (LT), Increased Bowman’s space (IBS), Degenerated glomeruli (DG), Infiltration of lymphocytic cells (IL), Degeneration (D), Hemorrhage (H), H&E stained—40×.

Histological alterations in three major organs (gills, liver, and kidney) were documented after 7 dpi in control and in *P. aeruginosa*-, and *F. oxysporum*-challenged groups. Histological examination revealed negligible changes in control fish while clear histopathological alterations were visible in *P. aeruginosa-* and *F. oxysporum*-challenged fish. Microscopic analysis of gill tissue morphology displayed quite an intact structure for control fish (Figure 5A). *P. aeruginosa-*infected fish showed alternations such as fusion of primary and secondary lamella, hemorrhage, shortening of lamellae, and hypertrophy of gill epithelial cells, but in the case of *F. oxysporum*-infected fish, they showed higher alterations in fusion of primary and secondary lamella, leukocyte infiltration, hemorrhage, tissue debris, and cellular necrosis (Figure 5B,C). Microscopic analysis of liver tissue morphology showed minor changes with the normal cellular organization of hepatocytes in control fish (Figure 5D). However, congestion of the hepatic portal vein, ruptured hepatocytes, and presence of vacuoles were observed for the *P. aeruginosa-*challenged group (Figure 5E). *F. oxysporum*-challenged fish showed discrete morphological alterations in the liver tissue architecture such as hepatic abscess, cytoplasmic degeneration, and leukocyte infiltration (Figure 5F). Control fish kidneys showed precise functional parts such as a nephron with glomerulus, bowman’s capsule, distal tubules, and proximal and collecting tubules with intact structures (Figure 5G). On the other hand, in *P. aeruginosa-*infected fish, damaged glomerulus, complete necrosis and hyperplasia of renal space, congested blood vessels, presence of infiltered leucocytes, hemorrhagic bowman capsules, and inflamed excretory tubules were seen. The *F. oxysporum*-challenged group had higher alternations in comparison to the control group (Figure 5H,I). Overall examination of tissue histology of gills, liver, and kidney revealed that the *F. oxysporum*-challenged group had higher alternations in comparison to the *P. aeruginosa*-challenged group.

### 2.6. Organ Index Analysis

The cumulative high organ index (HAI) showed that the extent of gill damage was higher in the *F. oxysporum*-challenged group in relation to the *P. aeruginosa*-challenged and the control groups (Table 2). Similarly, kidneys showed a HAI in the *F. oxysporum*-challenged group relative to the *P. aeruginosa* and control groups. Overall, the histological data revealed that the histological deformities index in the *F. oxysporum*-challenged group was higher and was in the order of kidney > liver > gills. In the case of the *P. aeruginosa*-challenged group, the order was liver > kidney > gills.

## 3. Discussion

In response to the increasing incidence of infections in aquatic species, we established an in vivo *C. mrigala* model to investigate the progression of the disease and the host’s immune response. Currently, *P. aeruginosa-* and *F. oxysporum-*infected fish show non-interactive and non-responsive behavior such as disturbed swimming orientation and irregular respiratory motions. In a previous study, abnormal behavior and mortality was also noted in *Channa punctatus* fish challenged with *P. aeruginosa* [20]. In another study, behavior and clinical changes, e.g., exophthalmia and abnormal swimming pattern were noted in *O. niloticus* challenged with *F. oxysporum* [21]. After *P. aeruginosa* and *F. oxysporum* infection in fish, several clinical changes appeared, e.g., skin/mouth ulceration, scale loss, tail/fin rot with skin discoloration. *Labeo rohita* (*L. rohita*) infected with *Aeromonas hydrophila* (*A. hydrophila)* also exhibited external symptoms of infection progression, i.e., discoloration of gills (pale red), blackening of descaled areas, sluggish movement, and lethargic eyes [22]. Amrevuawho et al. [23] also investigated external signs of infection in *Clarias gariepinus (C. gariepinus)* fish infected with *P. aeruginosa.* Fusarium infestation leads to severe infection in the form of epidermal lesions and highly localized skin defects, e.g., ulcer [24].

Proinflammatory cytokines have significant roles in protecting against microbial pathogens. *TNF-α* and *IL-1β* display bimodal patterns of stimulation recognized as essential factors for recruiting phagocytes and initiating the immune response at the infection site [25,26]. A scarcity of scientific literature exists regarding the expression of inflammatory genes in a diverse range of fish organs. Expression of *TNF-α* was increased significantly in the blood, livers, and kidneys of infected *C. mrigala. TNF-α* expression was significantly upregulated in blood of the large yellow croaker after 2 and 4 dpi with infection with *Vibrio parahaemolyticus* [27]. *TNF-α* expression was found to be upregulated in head kidney leucocytes after triggering with lipopolysaccharide [25]. These differences in expression might be associated to different fish species, infection time, and microbe type. Expressions of *TNF-α* in the gills of infected fish was increased. A weak regulation of the *TNF-α* gene was found in gills of *O. mykiss* fish challenged with *I. multifiliis* [28]. We found that *IL-1β* was upregulated in gills, livers, and kidneys of *P. aeruginosa-* and *F. oxysporum-*challenged groups but was downregulated in blood. *IL-1β* expression was significantly upregulated in heads, kidneys, and spleens of the Japanese flounder when exposed to *N. seriolae* [29]. 

*P. aeruginosa* and *F. oxysporum* infections led to variation in hematological indices of fish. Significant reduction of TECs, Hb, HCT, and MCV levels may cause erythrocytic anemia in fish, which becomes a major reason for infection progression and mortality in fish. Such erythrocytic anemia in a variety of fish spp. has been reported by Shah [30]. *S. parasitica and Pseudomonas putida* infections in tilapia cause a decrease in total number of RBC and Hb [31,32]; similar findings were obtained in the current study. The increasing trend in TLC counts in *P. aeruginosa-* and *F. oxysporum-*challenged fish could be due to deep tissue necrosis as shown by histological data. Infection and various stress-related factors cause tissue damage and cellular necrosis, which lead to an increase in granulocytes [30].

Biochemical analysis has significance in examining altered enzyme activity produced by a foreign toxicant. A rapid increase in ALT and AST activity is a chief indicator of hepatic dysfunction. Pathogenic infections elevate the release of liver enzymes by hindering their normal activation, as the liver exports these enzymes into the bloodstream in fish [33]. Current findings reveal that liver enzyme activities were significantly different between the infected group and the control group. Increased ALT, AST, and ALP enzyme activity in *L. rohita* infected with *A. hydrophila* was previously reported [22]. Significant variations in serum ALT, AST, and ALP were reported after fungal (*C. albicans*) infection in *O. niloticus* [34]. A decrease in serum protein level was found in *P. aeruginosa-* and *F. oxysporum-*challenged fish. A significant reduction in TP has been reported in *Saprolegnia*-challenged fish [35]. A decreased level of serum proteins is directly related to the infectious state and impairment of the synthesis process along with a low absorption rate and high rate of protein loss. Current findings reveal that Alb level significantly increased in the infected group. Similar alterations were reported in a previous study, in which the level of albumin was increased in *Paralichthys olivaceus* infected by *Vibrio scophthalmi* [36]. Thus, propagation of bacterial and fungal infection in fish induces physiological stress that ultimately causes overall hemostatic imbalance.

Histopathological alterations are good biomarkers for valuation of organ physiology. Major histopathological changes were observed mainly in gills, livers, and kidneys of *P. aeruginosa-* and *F. oxysporum-*infected fish. Saharia et al. [37] noticed clubbed and filamentous gill lamella, gill hyperplasia, congestion of the hepatic portal vein, and hepatocytes along with complete necrosis of renal tubules in *L. rohita* infected with *A. hydrophila.* The gill becomes an open target organ for initial toxicity from pathogenic strains, which results in erosion and reduction in respiratory space and buildup of pathogen attack leading to tissue hypoxia and eventually organ damage [38]. Abdelhamed et al. [39] noted gill hyperplasia, loss of branchial epithelia, lamellae degeneration, liver necrosis, and extensive stomach and intestine inflammation in catfish infected by *A. hydrophila.* The liver undergoes many destructive changes due to toxins and extracellular products released by bacteria. Entry of these toxic substances into hepatic portal vein sinusoid cells causes cellular necrosis, stretching of hepatocytes, vacuole formation, and inflammation [40]. Hyperplasia in gill secondary lamella, hyperplasia in lymph follicles of the spleen, hyaline degeneration, edema in the liver, and glomerular epithelium atrophy were caused by *A. hydrophila* in *C. gariepinus* [41].

When fish are challenged by pathogens, their immune systems launch a defense response to fight off the infection. Infections can disrupt the balance of cytokines (*TNF-α* and *IL-1β*), serum proteins, and white blood cell functions, all leading to inflammation and disease progression [42,43]. 

## 4. Materials and Methods

### 4.1. Experimental Fish Rearing and Maintenance 

A total of 135 juveniles of *C. mrigala* (22 ± 3.0 g body weight; 15 ± 2.0 cm body length) were obtained from Manawa Fish Hatchery, Lahore, Pakistan and transported to an aquaculture and fisheries laboratory. Fish were acclimatized to the local conditions for 14 days. Water quality indices were maintained as follows: pH (7.21 ± 0.82), temperature (26.0 ± 1.32 °C), dissolved oxygen (5.36 ± 0.81 mg/L), and ammonia (1.92 ± 0.04 mg/L) during the experimental period. Fish were fed with commercial diet at the rate of 3% of their body weight. A total of 30% of the water was exchanged on a daily basis, and fecal wastes were removed. The research was carried out in compliance with the animal welfare guidelines of the Institutional animal ethics committee of Lahore College for Women University, Lahore, Pakistan (protocol code: Zoology/LCWU/123; dated: 17 March 2021).

### 4.2. P. aeruginosa Culture Preparation 

*P. aeruginosa* culture was prepared in 10 mL of nutrient broth (HiMedia Ltd., Lahore, Pakistan) for the challenge trial. The culture was vortexed and then incubated in a shaker incubator at 37 °C for 24 h. After 24 h, the bacterial culture was centrifuged for 15 min at 3000 rpm to obtain the hard pellet, and the pellet was resuspended several times in sterile PBS (pH 7.2) for washing. Afterward, the optical density was estimated using a spectrophotometer at 600 nm, and the culture was diluted with PBS to a correspond concentration of 1.8 × 10^5^ CFU/mL.

### 4.3. Fungal Culture Preparation

*F. oxysporum* hyphae were inoculated onto triplicate malt extract agar (MEA CM059B, HiMedia Ltd., Lahore, Pakistan) plates and incubated at 28 °C for 9 days. A purified fungal culture was obtained through three successive sub-culturing steps on MEA plates. For spore suspension preparation, 1 mL of sterilized distilled water was added to the *F. oxysporum* culture plates, and the plate surfaces were gently scraped using a sterile spatula. The resulting suspension was transferred to a 50 mL falcon tube, vortexed to ensure thorough mixing, and diluted to approximately 10 mL with sterilized distilled water. A 10 μL portion of the homogenized spore suspension solution was placed on a hemocytometer and covered with a coverslip, and spores were then counted under a microscope at 40× magnification. The number of spores was determined using the following formula [44].

$$Number\;of\;spores\;per\;cubic\;mm\;suspension=\frac{Number\;of\;spores\;counted}{Number\;of\;smallest\;square\;counted\times 4000}\times dilution\;factor\;$$

### 4.4. Experimental Design

Fish were randomly divided into three major groups in triplicates (n = 15 in each group), i.e., the control and *P. aeruginosa-* and *F. oxysporum*-infected groups. Infected groups were intramuscularly injected with *P. aeruginosa* 100 µL of 1.8 × 10^5^ CFU/mL and *F. oxysporum* 1200 spores/0.5 mL/fish. The control group was injected with an equal volume of 1% sterilized PBS. Fish were monitored daily in order to observe any gross pathological symptoms appearing on the fish. Thereafter, at 7-days post-infection (dpi), fish were anesthetized in water containing 100 μg/L clove oil and sacrificed to collect blood and tissue (gills, liver, and kidney) samples. The blood sample was centrifuged at 3000 rpm for 5 min to separate the serum. The supernatant was then immediately transferred into a new tube and stored at −25 °C for further analysis. 

### 4.5. Cytokine Analysis

Total RNA was extracted by using the GeneJETTM RNA-Purification Kit (Catalog number: K0732) (Thermo Fisher Scientific, Waltham, MA, USA). The concentration of RNA was evaluated by measuring the absorbance ratio at 260 nm. The purity of the samples was analyzed using the nanodrop ND-1000 (Thermo Fisher Scientific, Waltham, MA, USA) by calculating the OD ratio at 260 nm/280 nm. To synthesize cDNA, the Thermo Scientific RevertAid First Strand cDNA synthesis kit (Thermo Fischer Scientific Inc., Waltham, MA, USA, Catalog number: K1622) was employed. Initially, 2 μg mRNA and 1 μL random hexamer primer were mixed with nuclease-free water to obtain a final volume of 12 μL. Then, 4 μL of 5× reaction buffer, 1 μL of Ribolock RNase inhibitor, 2 μL of 10 mM dNTP mix, and 1 μL of 200 U/μL RevertAid M-MuLV RT were added to the solution. The reaction mixture was incubated at 25 °C for 5 min and at 42 °C for 60 min. Subsequently, the reaction was terminated by heating it to 70 °C for 5 min. The resulting cDNA was stored at −20 °C until further analysis. For RT-qPCR (PikoRealTM Real Time PCR-System), the reaction included 1 μL cDNA, 7 μL Maxima-SYBR Green, and 1 μL of gene-specific forward and reverse primers (Table 3). The reference gene used was β-actin, and the ΔΔCt method was used to calculate the variations in gene expression (fold changes) [45].

### 4.6. Hematological Studies

Blood was collected from the caudal vein in EDTA tubes for the hematological analysis. Total erythrocyte count (TEC), hemoglobin (Hb), hematocrit (Hct), mean corpuscular volume (MCV), mean corpuscular hemoglobin (MCH), mean corpuscular hemoglobin concentration (MCHC), total leukocyte count (TLC), and differential leukocyte count (DLC) were analyzed using the automated hematological analyzer (Sysmex, KX-21, Kobe, Japan). 

### 4.7. Biochemical Analysis 

Enzymatic activities of alanine aminotransferase (ALT), aspartate aminotransferase (AST), and alkaline phosphatase (ALP) were quantified using specific kit protocols devised according to the International Federation of Clinical Chemistry and Laboratory Medicine method (IFCC). Serum total protein (TP) was measured using the Crescent diagnostic kit, Cat. No. CS.610. (Jeddah, Saudi Arabia), which employs the Biuret method. The albumin (Alb) level was assayed using the bromocresol green (BCG) dye binding technique using an Albumin kit (ANMOL-LAB Pvt. Ltd., Lahore, Pakistan. LOT. DR379E249). Globulin (Glob) was estimated by deducting the TP from albumin. The A/G ratio was obtained by dividing Alb from Glob. 

### 4.8. Histopathological Studies 

Tissue samples (gills, liver, and kidney) were preserved in 10% formalin (neutral buffer) to prevent cellular autolysis. Dehydration of fixed tissues was performed through differential grades of alcohol in ascending order for 15–20 min and then put in two rounds of pure xylene (100%) and embedded in paraffin wax. The samples were sectioned (6–7 μm thickness) using a rotary microtome (ERM-2301), mounted with DPX, and stained with Hematoxylin and Eosin. Photographs of the samples were taken under optical microscope (Nikon Eclipse E-200 Trinocular, Tokyo Japan Eil-12) to compare differences in tissue cell morphology.

### 4.9. Statistical Analysis

Data were expressed as mean ± SEM. Graph-pad prism software (Version: 4.0, San Diego, CA, USA) was used for statistical analysis of data. An analysis of variance (ANOVA) with Tukey’s multiple comparison test was performed to calculate the differences between groups. Results with *p < 0.05* or lower were considered statistically significant.

## 5. Conclusions

This research demonstrated the adverse effects and invasion of *P. aeruginosa* and *F. oxysporum* infections in *C. mrigala* in a comparative manner. Severe hemato-biochemical and histological modifications clearly indicated the disruptive influence of *P. aeruginosa* and *F. oxysporum* infections in fish. The proinflammatory marker genes *TNF-α* and *IL-1β* showed differential expression in infected fish blood, gills, and kidneys as compared to the control. The downregulation of *TNF-α* and *IL-1β* genes may indicate aggressive virulent behavior of *P. aeruginosa* and *F. oxysporum,* which might have suppressed or delayed a ubiquitous immune response to a robustly developing infection. Further molecular analysis of detailed interaction of cytokines will provide a better understanding of the complete defense response of fish to different pathogens. Moreover, the information provided from this work may help to find novel measures for the control and regulation of disease outbreaks in aquaculture. These findings encourage extending our knowledge regarding early screening and diagnosis of fish diseases via infection biomarkers. 

## Figures and Tables

**Table 1:** Comparison of mean behavior attributes in *C. mrigala* (n = 15) challenged with *P. aeruginosa* and *F. oxysporum*.

Categories of Behavior	Pattern of Behavior	Control	*P. aeruginosa*	*F. oxysporum*
General activity	➢ Swimming activity	+	-	-
	➢ Hovering	+	-	+-
	➢ Inactiveness	+	-	-
Interaction	➢ Non-specific interaction	+	-	-
	➢ Fin movement	+	+-	-
Maintenance behavior	➢ Competitive foraging ability	+	+-	-

+ Sign shows = (no disturbance), +- Sign shows = (moderate disturbance), - Sign shows = (severe disturbance).

**Table 2:** Cumulative high organ index of control and *P. aeruginosa*- and *F. oxysporum*-challenged *C. mrigala* (n = 15) after 7 dpi.

	Control	*P. aeruginosa*	*F. oxysporum*
Gills	0.050 ± 0.028 ^a^	16.65 ± 0.638 ^b^	17.95 ± 0.232 ^c^
Liver	0.167± 0.145 ^b^	24.50 ± 0.248 ^b^	23.13 ± 0.478 ^c^
Kidney	0.052 ± 0.017 ^a^	23.00 ± 0.714 ^b^	28.33 ± 0.356 ^c^

Data presented as mean ± SEM. Different letters in the same column show significant differences (*p* < 0.005) in the groups.

**Table 3:** Primer sequences of the genes (TNF-α, IL-1β, and β-actin).

Gene Name	Primer Sequence (5′-3′)	References
*TNF-α*	FW: CTCAACAAGTCTCAGAACAATCAGG RV: TCCTGGTTCCTTCTCCAATCTAGCT	[46]
*IL-1β*	FW: ACCCCACAAAACATCGGCCAACC RV: TCTTCTCCATTTCCACCCTCTC	[47]
*β-actin*	FW: GACTTCGAGCAGGAGATGG RV-CAAGAAGGATGGCTGGAACA	[48]

## Data Availability

The data will be available from the corresponding author upon request.
